# Angiographic Coronary Spasm in a Case of Spontaneous Subarachnoid Hemorrhage Mimicking Acute Myocardial Infarction

**DOI:** 10.4021/cr269w

**Published:** 2013-05-09

**Authors:** Hung Yi Chen

**Affiliations:** Department of Cardiology, Taipei City Hospital-Heping Branch, No. 33, Sec. 2, Zhonghua Rd., Taipei City 100, Taiwan. Email: anigi426@ms24.hinet.net

**Keywords:** Coronary spasm, Coronary angiography, Subarachnoid hemorrhage, Acute myocardial infarction

## Abstract

Neurologic stunned myocardium after subarachnoid hemorrhage (SAH) has been evidenced. Clinical presentations manifested as ST segment elevation by electrocardiography (ECG), left ventricular wall motion abnormality by echocardiography, and abnormal cardiac markers. The pathophysiology remains controversial. Coronary artery spasm has been proposed as a possible mechanism. However, most SAH patients with ECG and echocardiographic findings suggestive of myocardial infarction were lacking of angiographic evidence of vasospasm. We present a case of 66-year-old man complained chest pain with transient conscious loss on the street. He was sent to our emergency room by witness with clear consciousness and electrocardiography showing prominent ST-segment elevation. Because chest tightness was complained, emergent catheterization was arranged immediately. Coronary angiography demonstrated a narrowing lesion on mid right coronary artery without atherosclerotic change on other site. He was successfully treated with primary coronary balloon angioplasty for the narrowing lesion. Then the patient was sent to intensive care unit for further care. His following ECG demonstrated sinus rhythm with ectopic beats without ST segment elevation. Unfortunately, he became irritable and deterioration of conscious level few hour later. Computer tomography revealed subdural and subarachnoid hemorrhage. Conservative treatment was suggested by neurological surgeon consulted. The clinical presentation of the SAH patient mimicked acute myocardial infarction and coronary spasm was evidenced by angiography. We report the case and review the articles.

## Introduction

Neurologic stunned myocardium after SAH has been reported with clinical manifestation as ST segment elevation mimicking acute myocardial infarction. Catecholamine related coronary spasm could be a potential mechanism. This was evidenced with normal coronary artery after SAH by autopsy initially. However, the hypothesis remained controversial because most SAH patients presented with acute ST segment elevation were lacking of angiographic evidence of vasospasm. Excessive myocardial catecholamine release in patients with SAH results in ECG abnormalities and contraction band necrosis is another hypothesis, and it is supported by experimental findings. We reported a case of SAH with electrocardiography showing prominent ST-segment elevation in inferior leads. Coronary angiography demonstrated a narrowing lesion on mid right coronary artery without atherosclerotic change on other site. After balloon angioplasty, the following ECG returned elevated ST segment to baseline. Finally, the patient was expired on the following day without further electrocardiographic change. Our case demonstrated the electrocardiographic abnormality could be explained by coronary spasm and evidenced by angiography. However, it was not related with clinical outcome. We report the case and review the articles.

## Case Report

**Figure 1:**
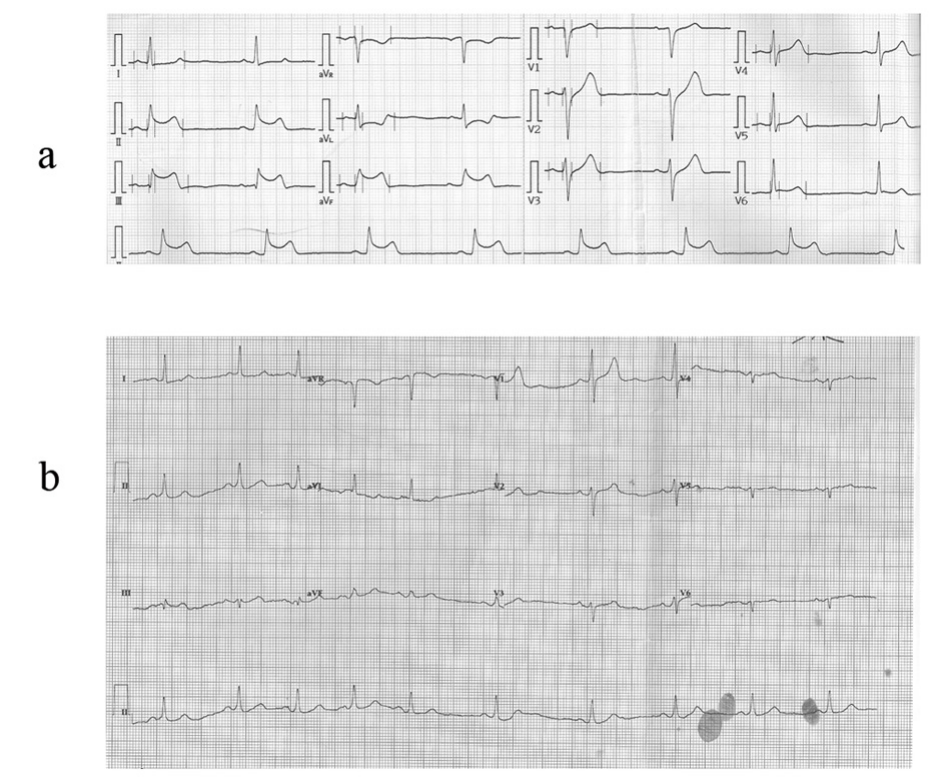
The electrocardiography demonstrated ST segment elevation on inferior leads II, III, aVF at emergency department (a) and without ST elevation after angioplasty (b).

**Figure 2:**
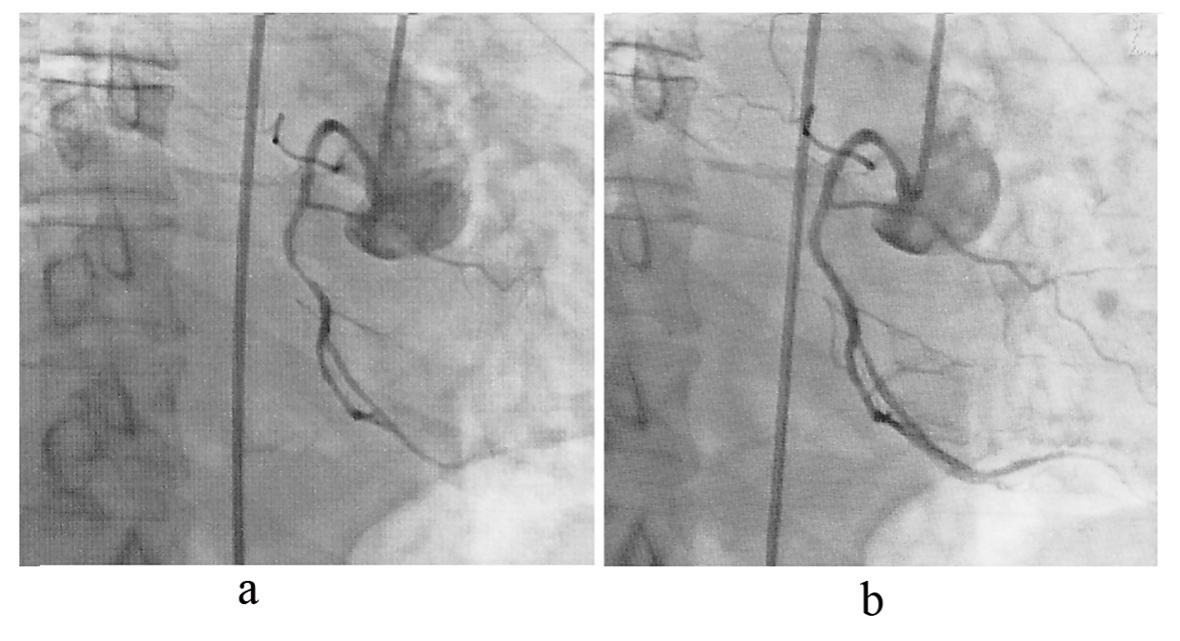
Coronary angiography demonstrated critical lesion over right coronary artery (a), and post-balloon angioplasty (b).

**Figure 3:**
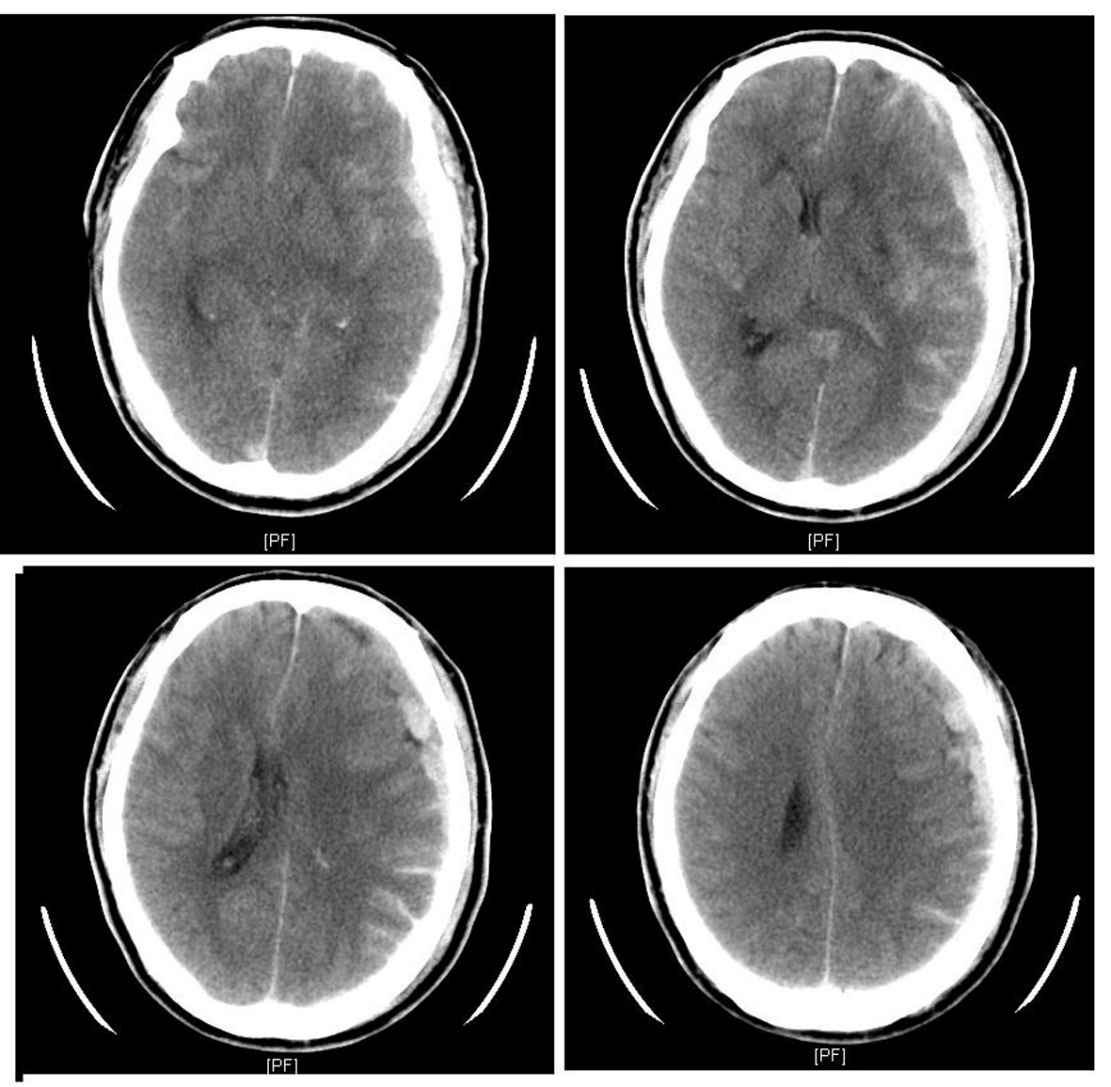
Head CT showed left subdural hematoma with subarachnoid hemorrhage and midline shifting to right side.

A 66-year-old man was admitted to our emergency department with complaining of transient conscious loss with spontaneous recovery when he was walking on the street and sent by witness. He had a history of hypertension and abdominal aortic dissection with medical control (amlodipine 5 mg and labetolol 200 mg twice daily). He is a heavy smoker. He was sent to our emergency department by ambulance where conscious recovery spontaneously and chest tightness complained. His electrocardiogram (ECG) revealed ST segment elevation on leads II, III, aVF (Fig. 1a). His blood pressure (BP) was 109/67 mmHg and pulse rate was 50 beats/min on arrival. Right-side ECG was checked for rule out right ventricular infarction. At our emergency department, the patient presents with slightly drowsy conscious and no mental impairment, otherwise, physical examination did not reveal any abnormalities as limb weakness. Chest radiography showed cardiomegaly with increasing interstitial markings. Biochemical analysis demonstrated slightly elevated cardiac enzyme (troponin I < 0.01 µg/L, total creatine kinase 232 U/L; reference range 30 - 170 U/L, and CK-myocardial isoenzyme 43 U/L; reference range 0 - 16 U/L) level. Under the impression of acute ST elevation myocardial infarction, emergency cardiac catheterization was arranged and it revealed obstructed lesion on mid right coronary artery (RCA) without significant stenosis nor atherosclerotic change on other site (Fig. 2a). After balloon angioplasty, the narrowing right coronary artery was re-opened with TIMI-3 flow and ST segment was turned down toward baseline by electrocardiographic monitor (Fig. 2b). However, ECG monitor remained frequent atrial and ventricular ectopic beats. The patient was sent to our intensive care unit for further treatment with stable hemodynamics. His following ECG revealed sinus rhythm without ST segment elevation (Fig. 1b). Few hours later, severe headache complained with concomitant progressive conscious disturbance. He received emergent computed tomography (CT) and showed subdural hematoma and subarachnoid hemorrhage (SDH, SAH) (Fig. 3). Neurosurgeon was consulted and conservative treatment was suggested. The patient was expired on the following day.

## Discussion

It had been reported that electrocardiographic ischemic change, abnormal elevation of cardiac enzymes, and even neurogenic stunned myocardium as segmental wall motion abnormalities may occur after central nervous system events as subarachnoid hemorrhage, subdural hematoma, and ischemic stroke [1]. The real pathophysiology remains unclear till now, coronary artery spasm, coronary thrombosis, and catecholamine induced oxygen supply-demand mismatch had been mentioned [2]. The hypothesis was supported by microscopic evidence of myocardial damage with normal coronary artery after subarachnoid hemorrhage by autopsy initially. In spite of coronary spasm had been proposed to be a potential mechanism, most patients with neurocardiogenic injury after subarachnoid hemorrhage presented with normal coronary artery and were lacking of angiographic evidence of coronary spasm [3-7]. Excessive myocardial catecholamine release in patients with SAH results in ECG abnormalities and contraction band necrosis is another hypothesis, and it is supported by experimental findings [8, 9].

In our case, initial prominent ST segment by electrocardiography and unstable hemodynamic suggested myocardial injury. Coronary angiography demonstrated right coronary artery narrowing lesion and the obstructed site was reopened after simple ballooning angioplasty. We believed it was vasospasm rather than atherosclerotic coronary artery disease by angiographic findings. After successful balloon angioplasty, the following ECG revealed no more ST segment elevation. This can explain his clinical picture manifested as acute myocardial infarction secondary to coronary spasm. However, although abnormal ECG and the elevated cardiac markers might be associated with coronary spasm, his clinical condition was not improved by successful balloon angioplasty. The patient was expired on the following day. It had been reported laboratory elevation of cardiac enzyme was associated with poor clinical outcome in those with subarachnoid hemorrhage [10]. Furthermore, our case showed the association can not explained by coronary spasm.

Electrocardiography findings with extreme prominent ST segment elevation could be consistent with a consequence of coronary vasospasm in subarachnoid hemorrhage and mimicked as acute myocardial infarction [2, 11]. Our case demonstrated coronary spasm in subarachnoid hemorrhage with sequential ECG finding and evidence by angiography. Finally, we emphasize subarachnoid hemorrhagic should be excluded in those present with extreme ST elevation suggesting coronary spasm, and we should keep in mind to prevent life-threatening tragedy, in spite of lacking significant clinical symptom and sign.
